# How Long Before a Second Defoliation of Actively Growing Grass Plants in the Desert Grassland?

**DOI:** 10.3389/fvets.2020.600734

**Published:** 2020-12-10

**Authors:** Sarah Noelle, Timothy Lyons, Alessandra Gorlier, Mitchel P. McClaran, Mary Nichols, George Ruyle

**Affiliations:** ^1^School of Natural Resources and the Environment, University of Arizona, Tucson, AZ, United States; ^2^Bureau of Land Management, Department of the Interior, Washington, DC, United States; ^3^Agricultural Research Service, Department of Agriculture, Washington, DC, United States

**Keywords:** stocking rate, adaptive management, regrowth rate, distance to water, grazing (rangelands)

## Abstract

In the Desert Grassland, second and subsequent defoliations on perennial grasses during the active growing season can have substantial impacts on grass recovery and reproduction following herbivory. Land managers implement tactics to avoid multiple defoliations on plants by way of rotational grazing, reduced stocking rates, and/or reduced time spent within a given pasture. We explored frequency and rate of defoliation by cattle on perennial bunchgrasses within an 11-day grazing period in three pastures including distance to water (300 and 600 m) and plant height to determine their influence on animal diet selection. Results indicate that 32% of all marked plants were defoliated by cattle and only 5% of defoliated plants were defoliated a second time by day 10 of the grazing period. Defoliation patterns in the studied pastures did not differ between two distances from water, or in relation to plant height. However, at the second defoliation cattle grazed plants that were shorter than at the first defoliation suggesting a selection for high quality re-growth over larger forage on offer. The results of this study show that a 10-day grazing period during the growing season of the Desert Grassland is an effective strategy to avoid second defoliations on individual perennial grass plants while maintaining sufficient forage for use during the dormant winter grazing season.

## Introduction

Avoiding a second defoliation on individual perennial grasses by livestock during the short 6–8-week growing season of the Desert Grassland has substantial benefits for sustaining and maintaining the condition of the available forage resource for future use (1–3). However, this type of management places limits on livestock production by forgoing the opportunity to graze high-quality summer biomass and particularly the high-quality regrowth of defoliated plants (4, 5). Understanding the rate at which grass plants receive second defoliations provides a basis for optimizing these tradeoffs for land managers (4, 6).

Second defoliations on a grass plant typically occur within 6–14 days of the grazing period, and before all plants within a pasture have been defoliated for the first time (5, 7–10). Livestock preference for re-growth has been related to the increased nutritional value of new regrowth and the removal of the less favored standing-dead biomass from previous growing seasons during the first defoliation (7, 11–14). The time between first and second defoliations is a function of the rate and amount of regrowth, and optimal grazing theory suggests that regrowth should approach a size equivalent to a full bite by the grazer (5). Moreover, a better understanding of the likelihood of repeated defoliation occurrence after cattle enter a pasture is needed since adaptive grazing management (15) aims to prevent multiple defoliations on perennial grasses by way of limiting grazing periods during the active growing season.

By applying these tenets to grazing management, questions are raised about how practical issues such as stocking rate and distance to water might influence the rate of first and second defoliations. These are relevant concerns in the Desert Grassland where typical pasture sizes commonly exceed 500–1,000 ha, and distance to water commonly exceeds 2 km.

Increased defoliation near water is typical because actual stocking rate (animals ha^−1^ unit time^−1^) increases as animals concentrate time spent in the center of the piosphere (16–22). However, there is some evidence that rates of first and second defoliations are not affected at modest (400 m) distances to water if livestock spend <12 days in a pasture (12). And even at low stocking rates, the interval between initial defoliations and successive defoliations have occurred anywhere from 6 to 10 days after the first defoliation (5, 7, 10).

To explore these relationships in the Desert Grassland, we monitored rates of first and second defoliations on perennial grasses as livestock moved into and stayed 11 days in three pastures during the summer growing season. Our monitoring of plant height provided opportunities to evaluate if first defoliations occurred on larger plants, and if plants that were defoliated a second time were smaller than when they were initially defoliated. Four livestock waters across the three pastures provided the opportunity to evaluate whether these patterns differed with distance to water. The primary objectives of this study were to (1) quantify the frequency of initial and repeat defoliation events on perennial grass plants, (2) determine the days to second defoliation after cattle enter the pasture, (3) examine if the height of plants at the second defoliation is equal to height at first defoliation, and (4) determine if distance from water affects the frequency and rate of first and second defoliations by cattle. The results of this study will provide land managers recommendations for timing of moving livestock between pastures in arid and semi-arid grazinglands to reduce the frequency of repeated grazing.

## Materials and Methods

### Study Area

This study was conducted in summer 2013 on the 21,500 ha Santa Rita Experimental Range (SRER) located at the northwestern base of the Santa Rita Mountains, ~50 km south of Tucson, Arizona, USA (31°50′31"N, 110°51′36"W). The SRER was established in 1902 and is among the oldest continuously operating rangeland research facilities in the world. Much of the long-term historical, ecological, and biological databases are available in digital form (23) on the SRER website (https://cals.arizona.edu/srer/).

The SRER ranges from 900 to 1,400 m in elevation and consists of gently sloping alluvial fans and some steep stony foothills and isolated buttes (24). Average annual temperature is 16°C with several nights of freezing temperatures in the winter and temperatures regularly exceeding 35°C in the summer. Rainfall is bimodal in distribution between winter (November-March) and summer (July-September) seasons, with average annual precipitation increasing with the elevation gradient from 275 to 450 mm (25).

Vegetation is characterized by desert grasslands dominated by short trees, primarily *Prosopis velutina* Wooton, shrubs, cacti, and other succulents, perennial grasses, and other herbaceous species. Perennial grass species include native species *Digitaria californica* (Benth.) Henr., *Muhlenbergia porteri* Scribn. ex Beal, *Aristida* spp. and *Bouteloua* spp. species, *Heteropogon contortus* (L.) Beauv. ex Roemer & J.A. Schultes, and *Setaria macrostachya* Kunth and non-native *Eragrostis lehmanniana* Nees (26).

The SRER has been continuously grazed by cattle since 1916 but until more recently, updates have been made to the livestock grazing management system to incorporate aspects of adaptive grazing principles by introducing a rotational grazing schema across the range. Currently, two herds of ~500 and 80 animals, respectively, move through the SRER's 38 pastures throughout the year. Livestock grazing management follows adaptive grazing principles to establish expected dormant season grazing capacity based on summer forage production, and summer grazing periods, 10-days in duration, based on avoiding the re-grazing of plants in the summer growing season (see Current Livestock Management Plan and Updates at https://cals.arizona.edu/srer/ and Comparison of Planned Livestock Use and Actual Use Since 2006 at https://cals.arizona.edu/srer/data.html).

### Experimental Design

During the 2013 short summer growing season (July to early September), a total of 800 marked perennial bunchgrass plants were randomly selected within three pastures of the SRER to quantify the timing and count of defoliation events during grazing (Table 1). At four livestock water sources across three pastures (one pasture with two waters), twenty 5 × 10 m plots were established; 10 plots at 300 m and 10 plots at 600 m from each water source. These distances from water were selected to avoid the expected greater use closer to water and to better represent use across the large pastures (800–1,900 ha). Water sources were set as replicates in the study and distance to water was included as an explanatory factor in the experimental design to assess its relationship with defoliation timing and frequency. Within each plot, 10 grass plants were randomly selected, for a total of 200 plants per water source. The five most common grass species in this 800-plant population were *M. porteri, D. californica, E*. *lehmanniana, S*. *macrostachya*, and *Aristida* spp. To facilitate relocation, each macroplot was georeferenced and the individual plants were marked with a 40-penny framing nail driven into the soil and with a small amount of pink flagging attached to the nail head. Previous studies on defoliation patterns indicated that this method of plant marking does not affect animal selectivity during grazing (9, 12, 27).

**Table 1:** Characteristics and management of the three pastures studied on the Santa Rita Experimental Range in summer 2013.

**Pastures and grazing periods**					**Herd size**	**Cumulative stocking rate of animal grazing days per hectare during grazing experiment (ADH)**				
**Pasture name**	**Size (ha)**	**Grazing period**	**Grazing days**	**Water sources**	**Animal Units (AU)**	**Day 1**	**Day 5**	**Day 10**	**Day 11**	**Day 15**
5S	1,902	06 July−16 July	11	2	494	0.26	1.30	2.60	2.86	–
5M	1,395	17 July−31 July	15	1	407	0.29	1.46	2.91	3.21	4.38
5N	819	28 July−11 August	15	1	432	0.53	2.64	5.27	5.80	7.91

To quantify the defoliation events during grazing, the height of all selected plants was measured at days 0, 1, 5, 10, and 11 of the days that livestock were in each pasture. Plant height was measured from the ground to the blade height of the tallest leaf (8, 9, 28). Reduced height between periods and the presence of tiller or leaf utilizations were used to indicate defoliation and categorize plants as undefoliated, defoliated, or re-defoliated. The timeframe and the frequency of observations were set based on studies suggesting that at low stocking rates the interval between initial defoliations and successive defoliations on individual plants is between 10 and 12 days, with the majority of second defoliations occurring from 6 to 10 days after their first defoliation (5, 7, 10). We quantified defoliation at 15 days of grazing for only two water sources (5M and 5N) because logistical issues prevented measurements at the other water sources. Because the sample size was only two waters, we limited our data presentation to simple mean and standard error of percent of plants defoliated once and twice over the 15 days.

We report grazing intensity during the short grazing periods (11–15 days) as both herd size during the start and end of the grazing period in each pasture, as well as the cumulative number of animal grazing days per hectare (ADH, Table 1). Cumulative ADH shows how grazing intensity increases for each day the herd continues to graze in a pasture, suggesting that the chances of defoliation on any plant increases through the duration of the grazing period.

Precipitation in July and August 2013 were drier than the long-term (1971–2019) average across the three pastures. The averages for July, August, and Jul-Aug for the combined values for the three closest (within or <200 m from the pastures) long-term rain gauges (gauges NW, DESST, and PAST3; see Precipitation at https://cals.arizona.edu/srer/data.html) were 69, 56, and 125 mm. Values recorded in July, August, and Jul-Aug 2013 and percent of long-term average were 39 mm (57%), 43 mm (76%), and 82 mm (65%), respectively. On 05 July 2013, there was a 30 mm rainfall event to start the summer growing season (see gauges 3 and 4 at https://www.tucson.ars.ag.gov/dap/DataCatalogueOld.htm).

### Data Treatment

The following data were summarized for each plant measured during the study: (i) height of the plants at days 0, 1, 5, 10, and 11, (ii) status of defoliation occurrence (defoliated vs. undefoliated); (iii) grazing day of each defoliation event (1, 5, 10, or 11); (iv) number of defoliations, (v) number of days between defoliations (1, 4, 5, 6, 9, or 10); and (vi) distance from water source (300 vs. 600 m).

For each grazing day (1, 5, 10, and 11), we calculated the percentage of plants undefoliated, defoliated once, and defoliated more than one time among total number of marked plants, as well as the percentage of plants grazed for the first or the second time among total number of defoliated plants. Using water sources as replicates, data from all plots at the same distance (300 or 600 m) from the same water source in each pasture were pooled together and count data were transformed to percentages for each replicate.

To assess the frequency of repeat defoliation events, we selected all plants defoliated two times and calculated the difference in days between the first and the second defoliation. For each resulting interval (i.e., 1, 4, 5, 6, 9, or 10 days), we calculated the relative frequencies of repeat defoliation given the number of plants available for second defoliation within that interval (i.e., plants already defoliated once).

### Statistical Analysis

The percentages of plants defoliated for the first or the second time at each grazing day were analyzed using Linear Mixed-Effects Models (LMMs) with a temporal correlation structure to account for the days of grazing as a time variable (29, 30). We focused separately on the occurrence of first and second defoliation events (i.e., response variables) to assess their variations over time and space. In both models, grazing days and distance to water were considered as explanatory variables, and the experimental units [4 replicates of water source, which avoids pseudo-replication, (31)] were set as random factors. The interaction between water distance and grazing days was also included in the models. Parameter estimation was based on the Maximum Likelihood (ML).

Before analysis, model assumptions were tested as suggested by Zuur et al. (30) on raw and transformed data. Logarithmic, square root, logit, and arcsine-square root transformations were tested. Although the model results did not differ, the logit transformation log{[(percentages/100) + 0.01]/[(1 – (percentages/100)) + 0.01]} (32) was selected for both first and second defoliation data as it better satisfied modeling assumptions and models showed the lowest Akaike Information Criterion [AIC; (33)] values. Finally, pairwise comparisons across factor groups were conducted applying the Bonferroni adjustment.

To assess the influence of plant height on the selection for defoliation, we first compared the height of the plants at the first defoliation with the height of the ungrazed plants. The analysis used a Two-Way ANOVA including defoliation (defoliated vs. undefoliated) and distance to water as fixed factors. A second analysis compared the heights of the same plants at the first and the second defoliation using a Mixed-Design Two-Way Repeated Measures ANOVA (Split-Plot ANOVA). Distance from water was included in the analysis and set as a between-subjects factor, while the count of defoliation events (first vs. second defoliation) and the interaction between time and water distance were set as within-subjects factors. Plants at the same distance from the same water sources were the subjects for the repeated statement. Because data were normally distributed and variances among groups were homogeneous (as tested with Shapiro-Wilk's and Levene tests, respectively), both Two-Way ANOVA and Split-Plot ANOVA were conducted on the raw data.

All statistical analyses were carried out on R statistical software 4.0.2 (34). The LMMs were conducted with the “lme” function (package: nlme) (35), while the Two-Way ANOVA and the Split-Plot Anova with the “aov” function (package: stats) (36).

## Results

Through 11 days of grazing, cattle defoliated 32% of the marked plants (255/800). Approximately 26% of plants were defoliated once (209/800), 43 plants (5%) were defoliated twice, and 3 plants were defoliated three times. Very few plants were first defoliated on day 1 (*n* = 10 of possible 800), but more than half of those (*n* = 7) were defoliated a second time by day 11. Many plants received the first defoliation from day 2 to 5 (*n* = 100), but only 36 (36%) of those were defoliated a second time by day 11. After 5 days of grazing, 13% of plants were defoliated one time and <1% of plants two times. After 10 days of grazing, 25% of plants were defoliated one time and 5% two times. Three defoliations on the same plant were observed only on day 11.

**Figure 1:**
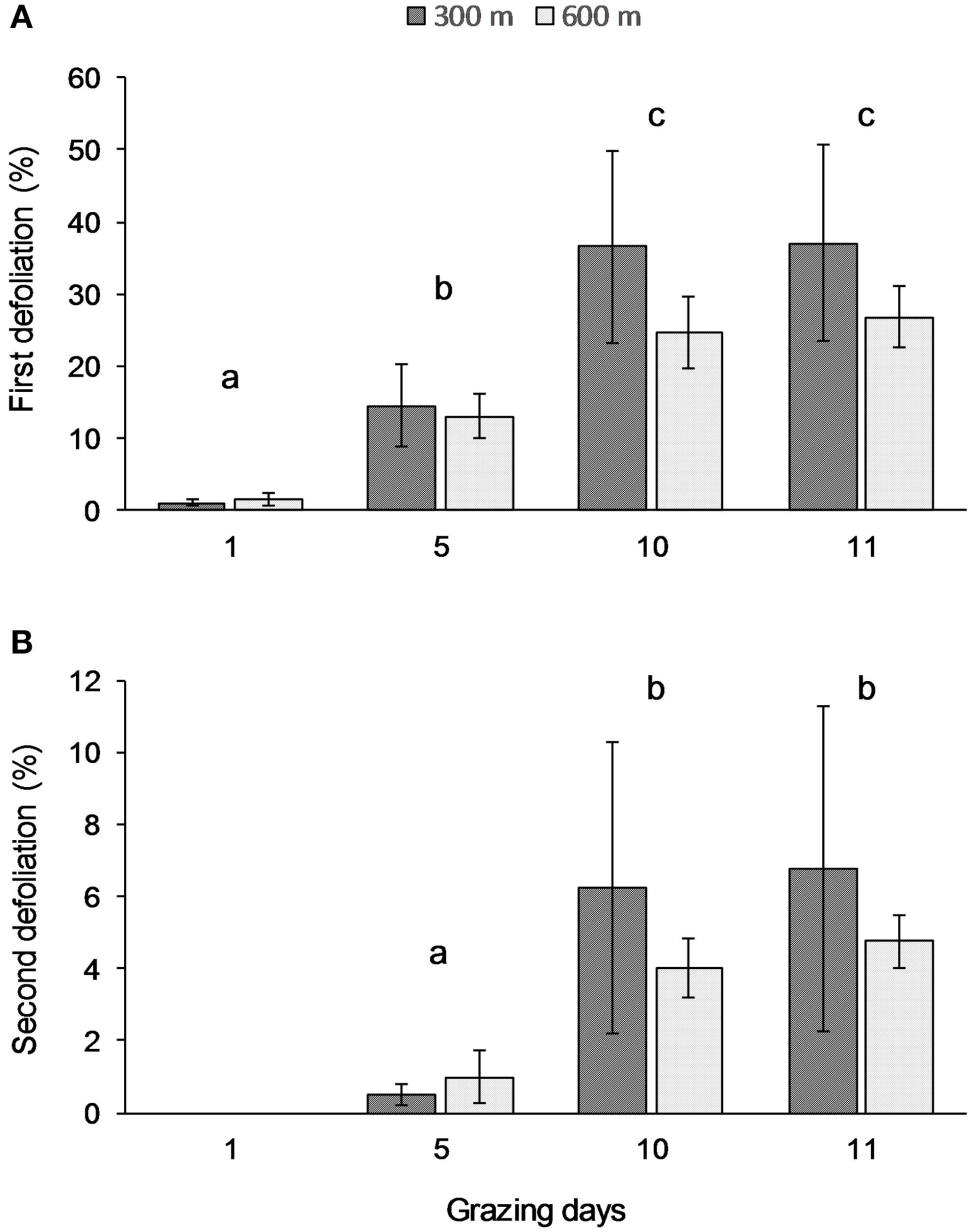
Average percentages of plants defoliated for the first **(A)** and the second **(B)** time at grazing days 1, 5, 10, and 11, respectively at 300 and 600 m from water sources. Error bars represent Standard Error of the Means. Lowercase letters indicate significant differences among days of grazing (**A**: *p* < 0.001; **B**: *p* < 0.01).

The percentage of plants defoliated for the first or the second time changed over the 11 days but was not related to distance from water source (Table 2). Specifically, the percentage of plants defoliated for the first time increased significantly (*p* < 0.001) between days 1, 5, and 10, while no changes were observed on day 11 (Figure 1A). Similar trends were observed of increasing frequencies of second defoliation events over time, but no relationship with distance from water source (Table 2). No plants were defoliated a second time on day 1, but the percentages of second defoliations varied significantly (*p* < 0.01) from day 5 to day 10 (Figure 1B).

**Table 2:** Results of the Linear Mixed-Effects Models (LMMs) showing the effects of distance from water sources, days of grazing, and their interactions on the occurrence of first and second defoliation events during grazing in three pastures.

**Factors**	**First defoliation**				**Second defoliation**			
	**(AIC** **=** **74.607)**				**(AIC** **=** **61.532)**			
	**df1**	**df2**	**F ratio**	***p*-value**	**df1**	**df2**	**F ratio**	***p*-value**
Distance from water	1	6	0.163	0.700	1	6	0.064	0.809
Days of grazing	3	18	40.018	<0.001	2	12	10.672	0.002
Distance from water x Days of grazing	3	18	0.434	0.731	2	12	0.068	0.935

*AIC, Akaike Information Criterion*.

For the two waters where defoliation was measured after 15 days of grazing, there was a trend of increased percent of plants grazed for the first and second time between day 11 and 15 at both distances from water (Table 3). Large variation in defoliation rates between the two water sources created high standard error values, but the trends suggest that the rate of second defoliations could exceed 15% of plants at 300 m from water.

**Table 3:** Average percentage of plants defoliated for the first and second time through 15 days of grazing at two distances from two water sources (5M and 5N).

**Distance from water**	**First defoliation (%)**					**Second defoliation (%)**			
	**Days of grazing**					**Days of grazing**			
	**1**	**5**	**10**	**11**	**15**	**5**	**10**	**11**	**15**
300	1.0 (0)	22.0 (9.0)	48.5 (24.5)	49.0 (25.0)	57.5 (30.5)	1.0 (0)	11.5 (6.5)	12.5 (7.5)	15.0 (9.0)
600	1.0 (0)	8.0 (1.0)	19.5 (7.5)	23.0 (7.0)	37.0 (18.0)	1.0 (0)	3.0 (1.0)	4.5 (1.5)	8.0 (4.0)

*Standard Error of the Means reported in parentheses*.

**Figure 2:**
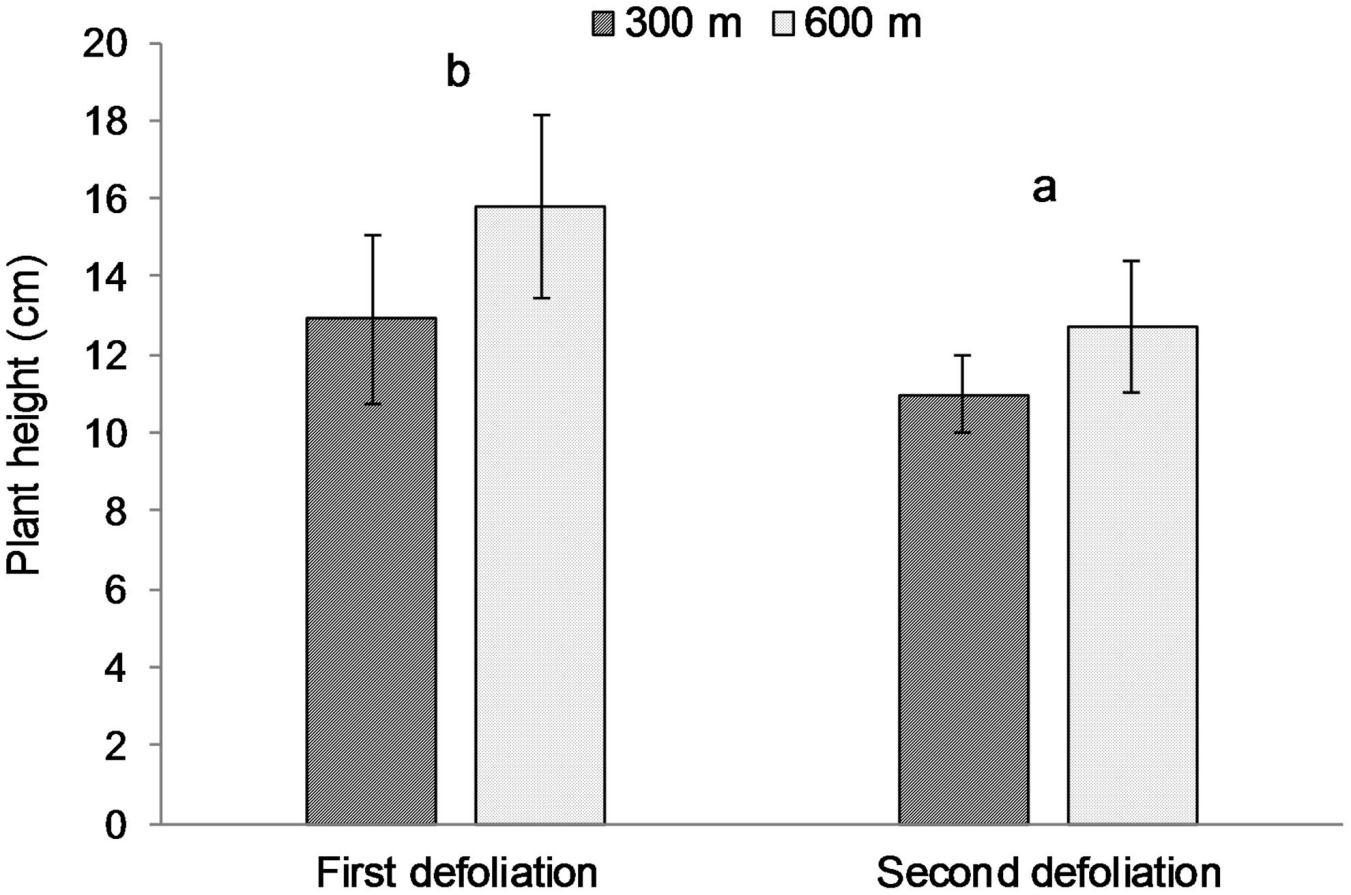
Average heights of the same plants at the time of the first and second defoliation, respectively, at 300 and 600 m from water sources. Error bars represent Standard Error of the Means. Lowercase letters indicate significant (*p* < 0.05) differences among defoliation time.

The average height of the plants (± Standard Error of the Means) at the beginning of the grazing period (day 0) was 6.86 cm (± 0.16) in pasture 5S, 17.42 (± 0.54) in pasture 5M, and 15.01 (± 0.46) in pasture 5N, respectively. The height of the plants defoliated for the first time at all days did not differ from the height of the ungrazed plants on those days (Table 4). However, the height of plants defoliated a second time was shorter than those same plants at time of first defoliation, and that difference was not related to distance from water source (Table 5). The Split-Plot ANOVA on plant heights confirmed that distance from water did not affect the timing of second defoliation events. Nevertheless, plant heights at the first and second defoliations differed significantly (*p* < 0.05) (Figure 2).

**Table 4:** Average height of the plants undefoliated and defoliated for the first time per grazing day.

**Days of grazing**	**Plant height (cm)**	
	**Undefoliated**	**First defoliation**
1	11.5 (1.8)	11.6 (4.8)
5	11.6 (1.5)	13.4 (1.2)
10	12.7 (1.5)	12.8 (1.6)
11	13.3 (1.4)	12.3 (1.3)

*Standard Error of the Means reported in parentheses*.

**Table 5:** Results of the Split-Plot ANOVA comparing the heights of the same plants at the first and the second defoliation and at 300 and 600 m from water (Average Heights with Standard Error of the Means reported in parentheses).

**Factors**		**Plant height (cm)**
Defoliation	*p*-value	0.037
	First defoliation	14.3 (1.6)
	Second defoliation	11.9 (1.0)
Distance from water	*p*-value	0.386
	300 m	11.9 (1.1)
	600 m	14.3 (1.5)
Distance from water x Defoliation	*p*-value	0.558

## Discussion

Without a doubt, avoiding a second defoliation during the short summer growing season will result in lost opportunities for livestock to graze the highest quality forage during the growing season. Our study indicates that by the 11^th^ day of grazing, ~5% of all plants had been defoliated twice, while only 30% of plants had been defoliated at least once. As a result, the cessation of grazing on day 11 will leave the majority of available perennial grass plants undefoliated and, unsurprisingly, these findings are very similar to previous work (7–9, 11, 37). A benefit of foregoing more use of summer growing season biomass is that the remaining biomass is then available for grazing as dormant winter season forage, during which plants are less susceptible to declines in vigor. However, the available mass in the dormant winter season is of lower forage quality than during the summer growing season (3, 5).

As we expected, at the four water sources, there was a substantial increase in the rate of second defoliations around day 10 of grazing with 5% of plants receiving a second defoliation by day 10 and did not decrease on day 11 (8, 9). Our observations at all four water sources during this study stopped at day 11 in order to more effectively evaluate the effects of water on second defoliations during the 10-day grazing period on the SRER. However, observations through day 15 at only two of the water sources provide an opportunity for informed speculation that rates of second defoliations will increase to as much as 15% or as little as 8%, at 300 and 600 m distance from water, respectively. These results suggest that the minimum length of a grazing period to avoid second defoliations should not be shorter than 10 days, and second defoliation rates after 15 days could be about 1 in 8 plants (15%) at 300 m from water. Shorter grazing periods may result in implementation difficulties in large pastures, maintaining animal performance, especially if cows are supporting young calves. As for longer grazing periods up to 15 days, there is no strong evidence that the rate of second defoliations would increase greatly if implementation problems were to delay a planned move to new pastures for a few days after day 10 of grazing.

Unexpectedly, plant height was not different between defoliated and undefoliated plants, suggesting that the forage mass on offer was not critical in the selection of rapidly growing plants during the short grazing period (37). However, plants defoliated a second time were shorter (11 cm) than when first defoliated (14 cm), suggesting that 11 cm of re-growth forage provides enough incentive with higher forage quality for cattle to re-visit previously grazed plants. In addition, it is widely thought that residual stems in bunchgrasses discourage grazing and cause livestock to preferentially select for taller plants with less old-growth material and longer leaf lengths (7, 12–14). The relatively dry conditions in July-August 2013 may have slowed the rate of regrowth following the first defoliation, and therefore delayed the time to second defoliation. However, the large amount of precipitation (30 mm) on 05 July provided very wet conditions to start the summer growing season.

Although distance from water is known to affect cattle distribution and grazing intensity (17, 21, 38), in our study, plant defoliation rates after 11 days did not differ between the 300 and 600 m distances from water. Of course, we would expect defoliations rates to be greater at 100 m than 300 m from water, but we focused at 300 and 600 m because they account for a much greater proportion of the pasture area (1.5–3% for 300 m and 6–13% for 600 m), than the <1% of the pastures at 100 m distance. The absence of a detectable difference in defoliation rates between 300 and 600 m may be due to a combination of environmental and management factors including that adapted cattle have been observed to travel on average 1.6 km from a water source while foraging (5) and move further from water especially when forage is limited or unattractive (39). Additionally, stocking rates are known to affect animal movements and grazing intensity. According to Bailey and Brown (5), under low stocking rates, cattle can travel further from water sources, while at high stocking rates they defoliate more intensively closer to water. We indeed observed higher variability among second defoliation events at 300 m (6.75 ± 4.52) than 600 m (4.75 ± 0.75) distance from water that can be attributed to the different stocking rates among pastures. However, a non-significant trend of greater defoliation rates at 300 than 600 m (71 and 29%, respectively) in the smallest pasture (5N) with the highest stocking rate suggests that even in short grazing periods, greater defoliations could occur closer to water if stocking rates are high (5).

The findings of this study have implications for land management in arid systems when goals of both conservative grazing and animal performance are important. For example, since 2006 management on the SRER has applied a 10-day limit to grazing use to avoid second defoliations in the short summer growing season (see Current Livestock Management Plan and Updates at https://cals.arizona.edu/srer/ and Comparison of Planned Livestock Use and Actual Use Since 2006 at https://cals.arizona.edu/srer/data.html). This management is applied with the purpose of sustaining plant vigor and to provide enough carry-over biomass to support grazing in the winter dormant season. The 10-day grazing period starts after receiving 1.25 cm of precipitation in July and ends when no new growth or flowering occurs typically in mid-September. The 10-day limit is applied to all pastures, whether they are small <200 ha or large >2,000 ha.

In practice, adhering to the 10-day limit is challenging for a variety of reasons including fence or water failures and when young calves slow the cow movement to new pastures. These delays have resulted in 14 or more days of grazing use in the summer months. Based on evidence from only two water sources, the rate of second defoliations could increase to an average of 15% at 300 m from water after 15 grazing days.

Further, the provision of large amounts of undefoliated and only once-defoliated plants for dormant season grazing has benefited the management at the SRER. Amount of dormant season biomass is determined in September and October for all pastures, and stocking rates are set to limit biomass utilization to 40% (see data on Grass Utilization by Livestock Since 2010 at https://cals.arizona.edu/srer/data.html) before the herd moves to the next pasture. In the end, the SRER has an adaptive grazing management program that (1) uses rainfall patterns each year to set the start and end of the conservative summer grazing season, (2) largely avoids second defoliations of plants in the short summer growing season by limiting grazing to 10 days, and (3) establishes the number of allowed animal grazing days/ha (varies from 3 to 18) to maintain utilization <40% based on the amount of summer biomass produced in each pasture.

## Data Availability Statement

The raw data supporting the conclusions of this article will be made available by the authors, without undue reservation.

## Author Contributions

SN provided primary authorship and leadership of this manuscript. TL designed and implemented the field experiment. AG conducted all data analyses and assisted with preparing the written manuscript. MM and GR served as Primary investigators for this study with contributions including assistance with project design, funding, statistical analysis design, and manuscript preparation. MN assisted with experimental design and implementation and provided manuscript review. All authors contributed to the article and approved the submitted version.

## Conflict of Interest

The authors declare that the research was conducted in the absence of any commercial or financial relationships that could be construed as a potential conflict of interest.
